# Doublet-Singlet-Doublet Transition in a Single Organic Molecule Magnet On-Surface Constructed with up to 3 Aluminum Atoms

**DOI:** 10.1021/acs.nanolett.1c02881

**Published:** 2021-09-14

**Authors:** We-Hyo Soe, Roberto Robles, Paula de Mendoza, Antonio M. Echavarren, Nicolas Lorente, Christian Joachim

**Affiliations:** † Centre d’Elaboration de Matériaux et d’Études Structurales (CEMES), Centre National de la Recherche Scientifique (CNRS), Université de Toulouse, 29 Rue J. Marvig, BP 94347, Cedex 31055 Toulouse, France; ‡ International Center for Materials Nanoarchitectonics (WPI-MANA), National Institute for Material Sciences (NIMS), 1-1 Namiki, Tsukuba, Ibaraki 305-0044, Japan; § Centro de Física de Materiales CFM/MPC (CSIC-UPV/EHU), Paseo Manuel de Lardizabal 5, 20018 Donostia-San Sebastián, Spain; # Institute of Chemical Research of Catalonia (ICIQ), Barcelona Institute of Science and Technology (BIST), 43007 Tarragona, Spain; ⊥ Department de Química Analítica i Química Orgànica, Universitat Rovira i Virgili, 43007 Tarragona, Spain; ○ Donostia International Physics Center (DIPC), 20018 Donostia-San Sebastian, Spain

**Keywords:** single-molecule magnet, spin multiplicity, aluminum−organic molecule complex, Kondo resonance, inelastic electron tunneling, LT-UHV STM

## Abstract

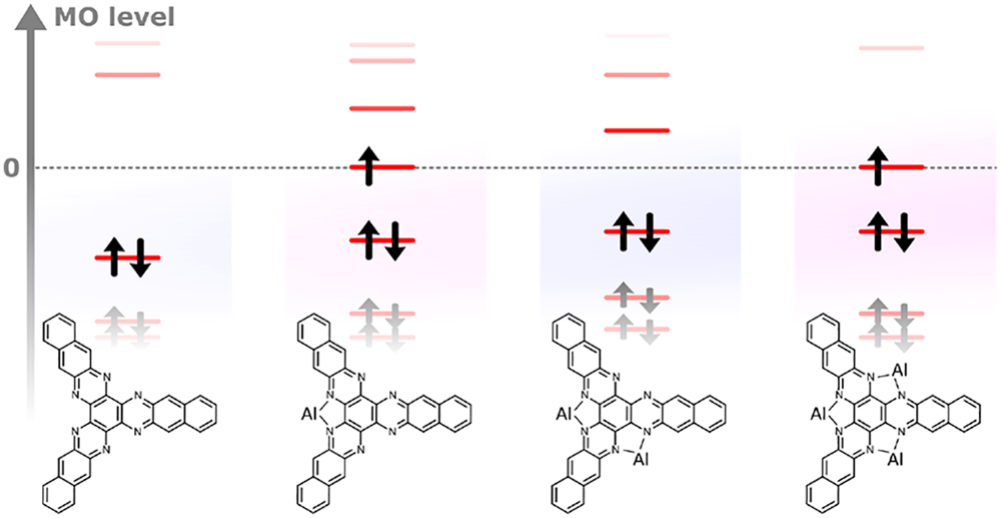

Starting from a long aza-starphene neutral and nonmagnetic organic molecule, a single-molecule magnet is on-surface constructed using up to 3 light nonmagnetic aluminum (Al) atoms. Seldom observed in solution with transition-metal atoms and going from 1 to 3 Al coordinated atoms, the doublet-singlet-doublet transition is easily on-surface accessible using the scanning tunneling microscope single-atom and single-molecule manipulations on a gold(111) surface. With 3 coordinated Al atoms, the lateral vibration modes of the Al_3_-aza-starphene molecule magnet are largely frozen. Using the Kondo states, this opens the observation of the in-phase Al vertical atom vibrations and out-of-phase central phenyl vibrations.

On-surface coordination chemistry uses various metals (Au, Ag, Cu, Fe, etc.) and ligands (cyano, isociano, pyridyl, carboxylic chemical groups), resulting in the formation of different coordination complexes as compared to solution chemistry.^1−3^ For example, a 3-fold coordination is typically encountered using a single Au adatom on a Au(111) metal surface,^4^ but not in solution.^5−7^ For a light nontransition-metal atom-like aluminum, we have recently demonstrated that its oxidation state on a Au(111) surface is between Al(I) and Al(II), leading to the on-surface formation of a magnetic molecule after coordinating two Al adatoms to a tetrabenzophenazine nonmagnetic molecule.^8^ Such light-atom magnetic molecule is not accessible in solution yet. Going a step further in intramolecular spin frustration and ordering, a doublet-singlet-doublet magnetic transition is also observed in solution for only a few organometallic complexes when coordinating not two but three d electron transition-metal atoms.^9,10^ We demonstrate here that this doublet-singlet-doublet transition can now be observed step-by-step by coordinating 1, 2, and 3 Al adatoms to a 1,8,9,16,17,24-hexaazatrianthracene (C_36_N_6_H_18_; HATA) long aza-starphene molecule also on the Au(111) surface. Furthermore, the coordination of 3 Al adatoms to HATA on a surface is freezing its lateral surface vibrational mode. Using the Kondo phenomenon, the on-surface Al_3_–HATA magnetic moment renders observable some of its very specific vertical vibrational modes like the in-phase 3 Al atoms vertical vibrations in the complex.

The HATA molecules were sublimated on a Au(111) UHV cleaned surface with a 583 K of sublimation temperature. Then, the sample was directly UHV transferred on the sample stage of our LT-UHV 4-STM, where each LT-STM is performing like a single LT-UHV-STM.^11^ When thermalized at liquid helium temperature (about 5.3 K in our setup^12^), single Al adatoms were evaporated through a small window opened in our cryostat in the UHV. This Al adatom source is simply a small ultrapure Al wire entwined in a tungsten filament coil. The tungsten filament is joule heated up to a yellow color during 60 s, and the produced Al atoms are guided using a little tantalum tube toward the Au(111) surface.

The sublimated HATA molecules are not only stabilized at the herringbone kinks of the Au(111) reconstructed surface but also attached to surface impurities on the terrace area without clustering. To study the electronic structure of a bare HATA molecule near the Au(111) surface Fermi level energy, single molecules attached on impurities were detached one by one by STM tip mechanical manipulation, i.e., reducing the STM tunneling junction resistance down to 15 MΩ. This STM manipulation protocol was also used to step-by-step construct the organometallic complexes with one HATA molecule and the deposited single Al adatoms. In this case, the STM tunneling junction resistance was below 50 MΩ in an STM “pushing” manipulation mode.^13^

Figure 1a–c presents a series of STM images resulting from the step-by-step construction of Al_*x*_–HATA complexes with up to *x* = 3 Al adatoms. During a single HATA STM tip molecular manipulation and reaching its first Al adatom, the HATA molecule systematically found a surface minimal potential energy location where this Al goes always in between two HATA anthracene molecular branches and in between the 2 nitrogens of the adjoining pyrazine molecular groups. Once the first Al is coordinated in between those 2 nitrogens, HATA can be easily STM manipulated, carrying this Al. This structural stability permits us to further coordinate one and two more Al adatoms to HATA as presented in Figure 1. Following the number of Al adatoms coordinated to HATA, 0, 1, 2, and 3 protrusions are observed around the Al_*x*_–HATA complex center on the STM images. Furthermore, the STM contrast at its center is increasing according to the number of coordinated Al adatoms.

**Figure 1:**
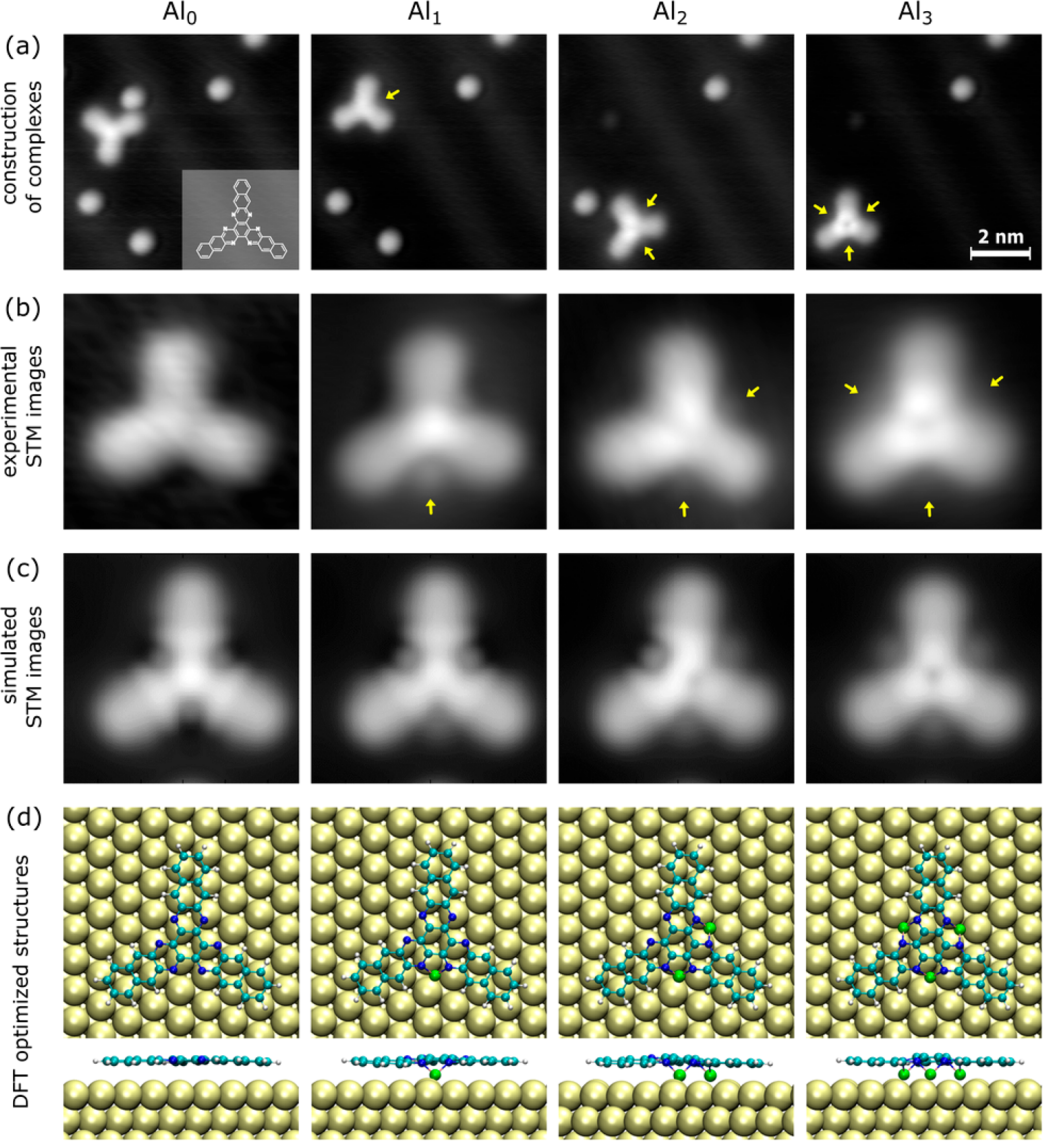
(a) From left to right, the one-by-one construction of the aluminum organometallic complexes, with up to three Al atoms on the HATA molecule. The STM images were recorded at a 500 mV bias voltage and 200 pA constant tunneling current intensity. The chemical structure of the hexaazatrianthracene (HATA) molecule is inserted in the leftmost image in panel a. (b) Experimental STM zoomed-in images of Al_*x*_–HATA molecules (*x* = 0, 1, 2, and 3 from left to right), where the single Al coordinated adatoms can be observed (yellow arrows). (c) The corresponding simulated STM images using the optimized HATA surface structures presented in panel d, resulting from DFT calculations.

To better understand the on-surface conformation of those complexes, DFT electronic structure calculations with geometry optimization were performed using the VASP code.^14^ PBE^15^ was used for the exchange and correlation functional, and the core electrons were treated using the projector augmented-wave method.^16^ The wave functions were expanded using a planewave basis set with an energy cutoff of 400 eV. For the Au(111) surface adsorption, the missing van der Waals interactions were included using the Tkatchenko–Scheffler scheme.^17^ The Au(111) surface was simulated using a slab of four layers in a 10 × 5√3 unit cell. The two upper layers, the molecule and the Al adatom positions were relaxed until all forces were smaller than 0.01 eV/Å. Relaxation was performed using the gamma-point sampling, while the properties of the electronic structure were calculated using a (3 × 3 × 1) k-point grid.

We have explored different geometries of the HATA molecule on the surface by varying its adsorption site and considering the anthracene arms oriented along [101̅] and [112̅] directions. The most stable configuration has the center of the molecule on top of an Au atom with the arms along the [112̅] direction. The closest configuration has almost degenerated with an energy difference of just 8 meV. In this configuration, the center of the molecule is in a hollow position of the Au(111) surface, and the anthracene arms are oriented along the [101̅] direction. Experimentally, the molecule is mainly found along this direction, so we will use this geometry in the discussion below. Following the experimental results, we have added 1, 2, and 3 Al atoms to the molecule in between the anthracene arms, and we have relaxed the atomic positions. The optimized structures are shown in Figure 1d. The Al atoms stay at hollow positions of the surface and are coordinated to the N atoms of the molecule. The corresponding Figure 1c STM images were simulated using the STMpw code.^18^

After constructing the Al_*x*_–HATA complexes one after the other, d*I*/d*V* tunneling spectroscopy measurements were performed on all those complexes including the bare HATA molecule. As presented in Figure 2, the STM tip apex was located where the d*I*/d*V* mapping is showing a large differential d*I*/d*V* conductance signal, i.e., at the HATA center, at the end of one of its anthracene branches, and at the Al coordination positions. For a voltage range above 500 mV (both polarities), d*I*/d*V* spectra of the bare HATA and of its Al_*x*_ complexes correspond to the tunneling resonances of its low-lying valence electronic states as provided in the Supporting Information.

**Figure 2:**
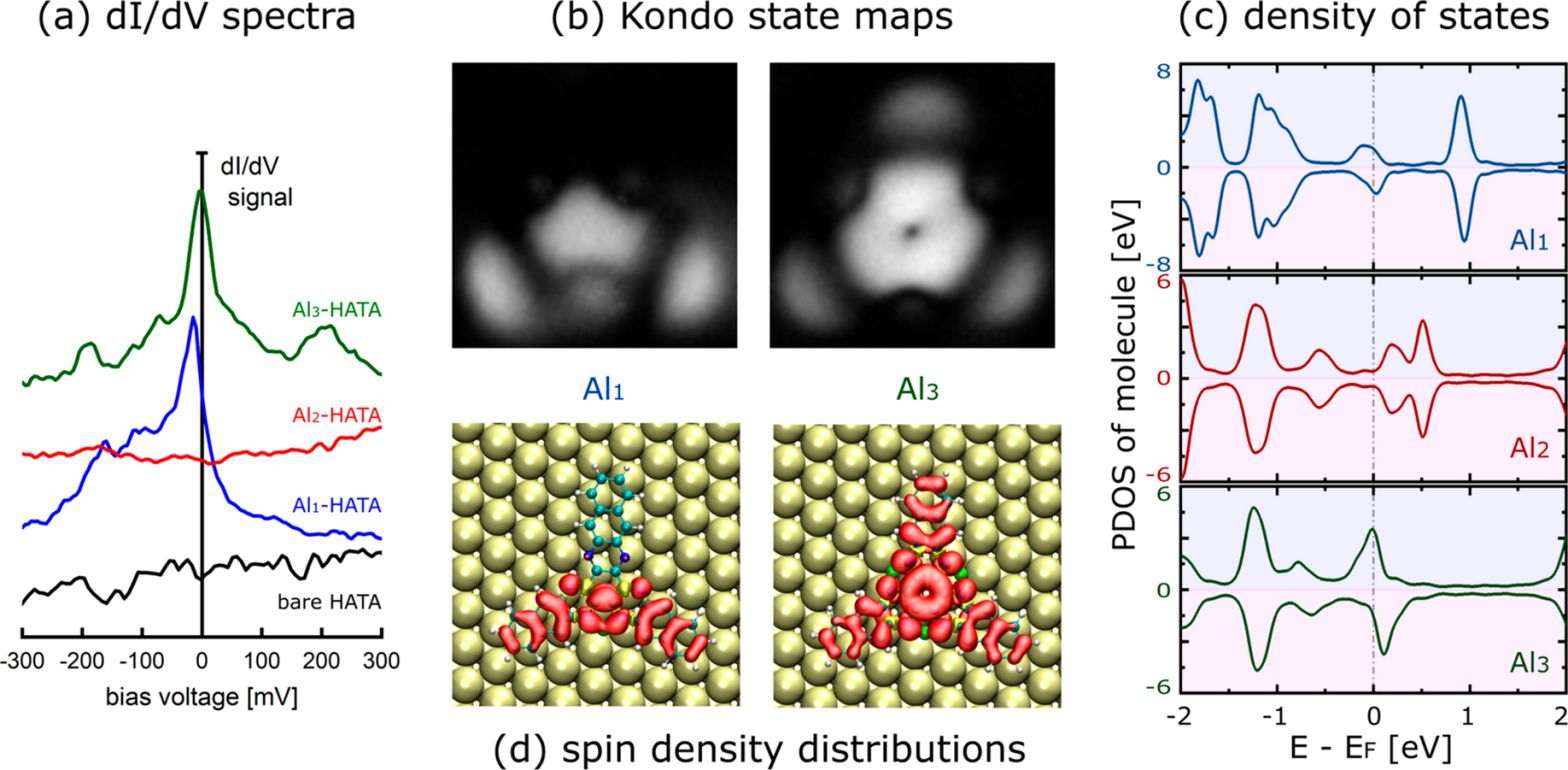
(a) The d*I*/d*V* tunneling spectra around the Au(111) surface Fermi level recorded by positioning the STM tip apex at the center of the molecule for each complex. For each bias voltage, those spectra were recorded using a lock-in amplifier with an 8 mV of voltage modulation. (b) The d*I*/d*V* differential conductance maps of the Al_1_– and Al_3_–HATA complexes recorded at −15 mV. Those conductance maps are presented contrast inverted because the −15 mV scanning bias voltage was set up slightly higher than the blue and dark-green Kondo resonance maxima in panel a to secure enough distance between the STM tip end apex and the HATA molecule. It fully reflects the state lying toward the low-energy region.^25^ (c) DFT calculated projected densities of states (PDOS) for the three HATA complexes showing the magnetic character of the Al_1_– and Al_3_–HATA complexes. (d) The DFT calculated spin density maps of the Al_1_–HATA and Al_3_–HATA complexes. Al_2_–HATA is not presented because of no spin polarization in this case.

Zooming now around the Au(111) Fermi energy, i.e., around zero bias voltage, d*I*/d*V* Kondo resonances were observed when positioning the STM tip apex at the center of the Al_1_ and Al_3_ complexes but not observed at the center of the bare HATA molecule nor at the center of its Al_2_ complex (Figure 2a). As already demonstrated on Au(111), even with no transition metal atom involved, the on-surface coordination of an aza-like aromatic molecule with Al adatoms is able to create noninteger spin states delocalized on the conjugated molecular core of this complex.^8^ Here, we went a step further, observing an on-surface doublet-singlet-doublet transition with no d electron transition metal atom involved. Such doublet-singlet-doublet transition is seldom observed in solution with organometallic transition metal complexes (see, for example, with hexaazatrinaphtynene (HAN)-based cobalt complexes^9,10^) because of the structural flexibility of those complexes in solution that plays against spin ordering.^9,10^ On-surface coordination chemistry is compensating this effect and gives access to other resonances as discussed below. With the d*I*/d*V* mapping around zero bias presented in Figure 2b, the spin-density distribution along the ligands can be unambiguously attributed to a Kondo-state. For example, the spin-density of the Al_1_ complex is distributed into specific molecular segments of the HATA molecule, i.e., at the center of the molecule and on the two anthracene branches enclosing this Al adatom, but nothing at the remaining anthracene molecular branch.

Looking at the electronic structure of the Al_*x*_–HATA complexes, we find that the Al_1_ and Al_3_ complexes are spin-polarized, as it can be observed on the DFT calculated projected densities of states (PDOS) (Figure 2c). This spin polarization can be understood by considering that each Al atom donates around two electrons, one to the molecule and one to the surface, such that each Al atom is in an oxidation state between Al(I) and Al(II) on the Au(111) surface.^8^ When the number of Al atoms is odd, the molecule gets an odd number of electrons, which gives rise to an unpaired electron and, therefore, to spin polarization. We have also plotted the spin density maps (Figure 2d), which nicely agree with the experimental d*I*/d*V* maps around zero bias voltage presented in Figure 2b. In particular, we observe that, for the Al_1_ complex, there is no spin density on the anthracene arm opposite to the coordinated Al atom. (See in the Supporting Information for how the spin density is distributed around Al atoms.)

For an Al_1_ complex, a variety of the Kondo–Fano resonance shapes can be observed. For example and as presented in Figure 3a, an asymmetric peak resonance ① is first observed on the HATA molecular core. Recorded on the same location on the molecule, it changes to the symmetric one ② after a small change in the adsorption conformation (or site) of the Al_1_ complex induced by an STM tip manipulation along the Au(111) surface. In addition, another STM manipulation leads to the new different asymmetric peak ③. For those Figure 3a resonances, the calculated Fano asymmetry *q* parameters^19^ are, respectively, −5, 40, and 2 going from ① to ③. Moreover, the Kondo–Fano resonances recorded on the HATA molecular core and at the Al location are clearly different as presented in Figure 3c,d. Symmetric peaks are observed on the core and asymmetric ones at the Al location (Figure 3c). When an asymmetric resonance is observed on the molecular core, the symmetric ones are detected at the Al locations (Figure 3d). Calculated *q* values in Figure 3c are 40 and −3 on the core and at Al locations, respectively, but 2 and 40 in Figure 3d. Those *q* values are characteristics of the coupling strength between the magnetic states and the metallic continuum (here, the Fermi sea of the gold surface). Since the Kondo effect is a screening effect of a magnetic impurity by conduction electrons, it includes a phase shift of the scattering process.^20^ The observed STM d*I*/d*V* Kondo resonances result from a coupling between tunneling electrons and this scattering electron cloud, whose character reflects the magnetic property of the impurity but also how it is adsorbed on the surface. Depending on the STM tip location, this large *q* difference is coming from the phase modulation of the tunneling electrons through the discrete Kondo state path.^21^

**Figure 3:**
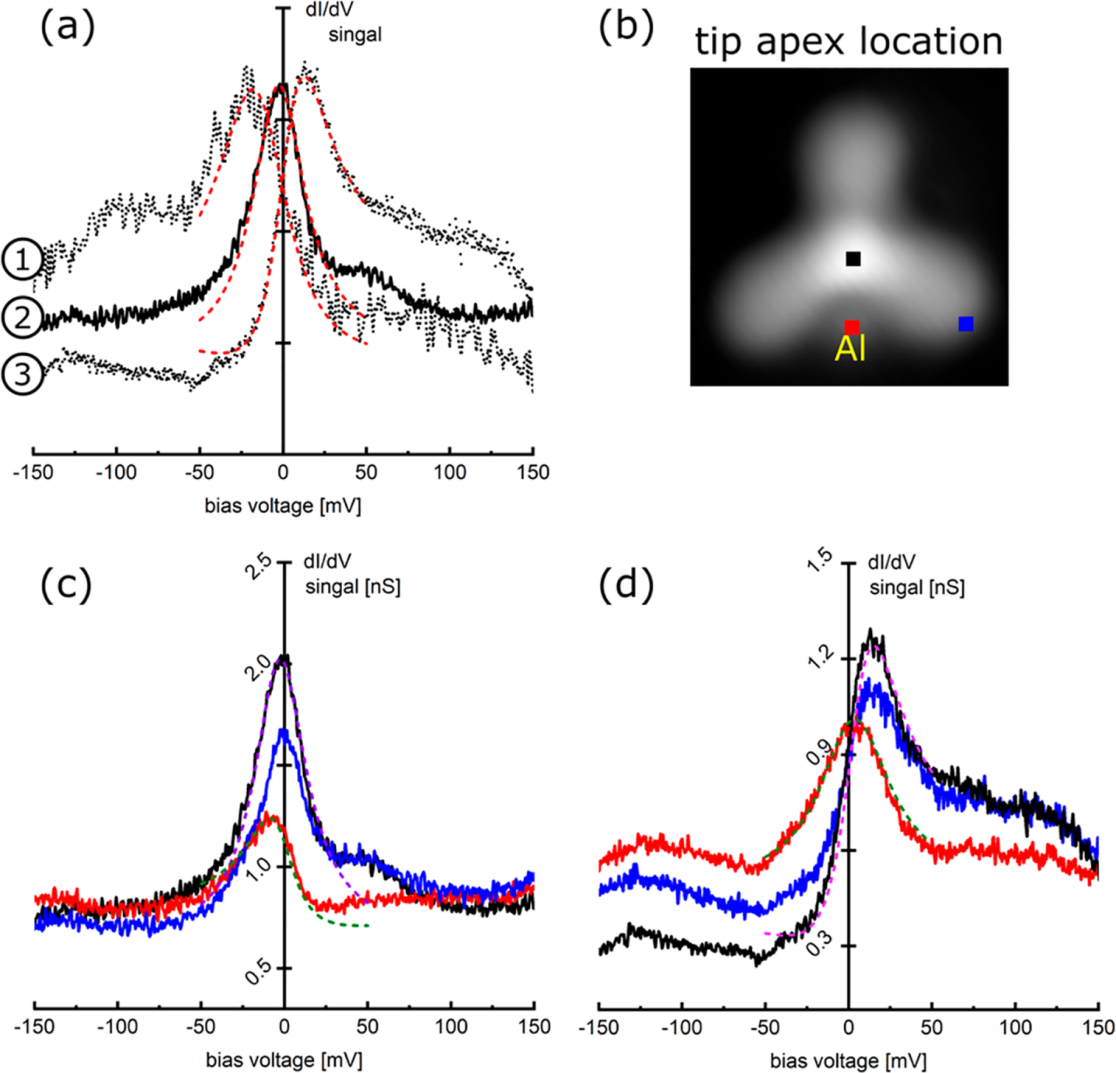
(a) The change of the d*I*/d*V* tunneling spectral shape (Kondo–Fano resonance) at the center of the Al_1_ complex before and after the STM manipulation of this complex. ① Asymmetric shape with a peak at the negative energy side (Fano asymmetry parameter *q* = −5) just after the construction of the complex. ② After STM manipulating this complex laterally on the surface, the resonance becomes almost symmetrical (*q* = 40). ③ Further lateral manipulations resulted in an asymmetrical shape with a resonance peak on the positive energy side (*q* = 2). After the change by those lateral manipulations of resonance peak shape, the d*I*/d*V* spectra were recorded at different tip locations indicated in panel b. The Kondo character of the resonances measured at the center of the molecule (black) and at the end of the anthracene branch (blue) is the same in panels c and d. However, the peak shape recorded on the Al sites (red) is different because of the change of the Al atom position. All smooth, dotted lines are the Kondo fitting based on the Fano theory.^19^

In the on-surface assembled Al_3_ complex, the HATA molecular core has no freedom to adopt different lateral surface conformations because, as compared, for example, to the Al_1_ complex, it is laterally rigidified by its three Al coordinated atoms. As a consequence, symmetric Kondo–Fano resonances are recorded around the central core of the molecule. In addition, two pairs of satellite peaks can also be observed as presented in Figure 4a. They are associated with the opening of inelastic tunneling channels coming from molecular vibrational modes.^22^ Generally, the opening of an inelastic tunneling channel creates a step function in the first derivative (d*I*/d*V*) of its tunnel junction *I*–*V* characteristics i.e., a Lorentzian like d^2^*I*/d*V*^2^ second derivative. Here, for the Al_3_ complex, the intensity of the d*I*/d*V* characteristics rises abruptly while increasing the bias voltage but goes down gradually instead of remaining of high intensity according to a step function. This inelastic tunneling feature is known as phonon-induced processes assisted by the Kondo state, instead of coming from the metal substrate bulk band.^23,24^ Since both inelastic tunneling phenomena occur due to the interactions of the molecular vibrations with the molecular complex Kondo state, the bare HATA molecule and its Al_2_ complex have no chance to open such new inelastic channels.

**Figure 4:**
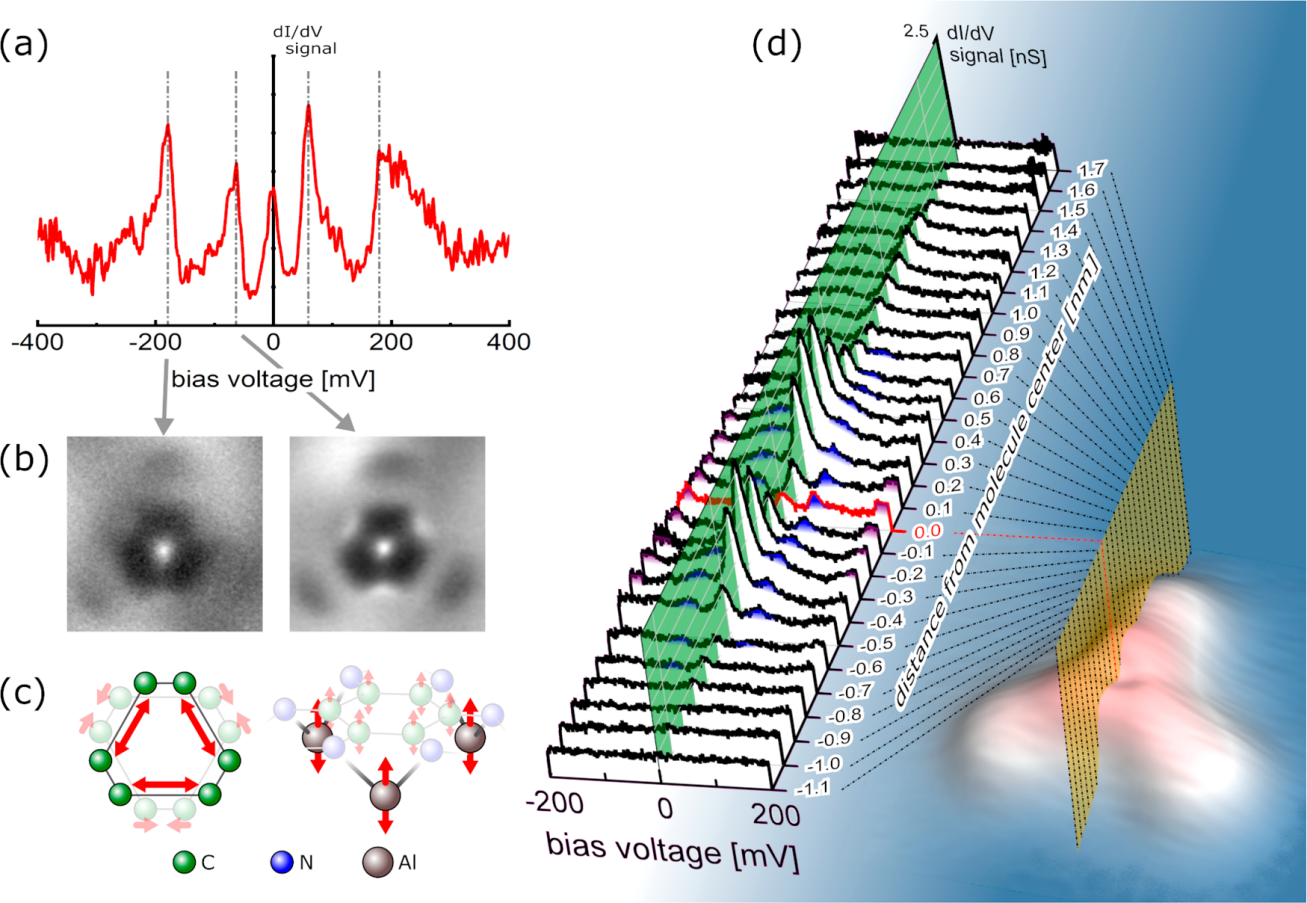
(a) The d*I*/d*V* spectrum exactly recorded at the center of the Al_3_–HATA molecular complex. (b) The differential conductance maps recorded at the energy of the two indicated different phonon channels. (c) The corresponding calculated vibrational modes at those energies. Each molecular vibration mode is collective motion, here, illustrated only with the corresponding core atoms. (d) A large series of spectra along the central line of the Al_3_–HATA complex. The low-energy phonon peak can be followed along from −0.7 to 0.7 nm relative to the center of the molecule (blue painted). The high-energy ones can be followed from −0.4 to 0.4 nm (purple).

To open both channels, the molecular vibration eigenmodes must be well-defined. This is not the case for the Al_1_ complex because its vibration modes are fuzzy due to the lateral motion of the HATA molecule stabilized laterally on the Au(111) surface only by one Al adatom. As presented in Figure 4d, both vibration eigenmodes resonances are located around the HATA phenyl central core because their resonance tail overlaps with the central electronic Kondo resonance. At the inelastic peak positions, differential conductance maps were also recorded. They are strongly perturbed by the Kondo resonance as presented in Figure 4b.^25^ However, it is still possible to observe the difference between the low- and high-energy vibrational eigenmodes, i.e., whether or not the Al adatom mechanical oscillations are deeply entangled with the HATA molecular vibrations. From the vibration eigenmode analysis using DFT calculations, the low-energy eigenmodes correspond to the Al adatoms and the phenyl central core oscillating perpendicular to the surface in the opposite phase. (A detailed analysis is provided in the Supporting Information.) The high-energy eigenmode corresponds to the phenyl ν_14_ mechanical mode.^26^

Going from one to three coordinated transition metal atoms in solution, observing a double-singlet-doublet magnetic transition in an organo-metallic molecular complex is generally difficult. The main reason is that spin ordering is generally overshadowed by the deformation of the molecular structure due to spin frustrations. On a surface, such a molecular structure flexibility is largely frozen. As a consequence, and using LT-UHV STM single atom and molecule manipulations, we have on-surface constructed step-by-step a single-molecule magnet where this doublet-singlet-doublet transition can be followed step-by-step using the Kondo effect on a Au(111) surface. With 3 coordinated Al atoms, we have also beneficiated from this structural freezing to record the inelastic signature of the in-phase Al vertical atom vibrations and of the out-of-phase central phenyl vibrations in the Al_3_ molecular complex.

## Supporting Information Available

Molecular electronic states; experiments vs DFT and vibration modes of the Al_*x*_–HATA molecule complexes (PDF)
DFT calculated low-energy eigenmode of the Al_3_ complex molecular vibration (MP4)
