# Supplementary material for: Doublet-Singlet-Doublet Transition in a Single Organic Molecule Magnet On-Surface Constructed with up to 3 Aluminum Atoms
Source: Nano Lett. 2021 Sep 14;21(19):8317–23. doi: 10.1021/acs.nanolett.1c02881 (PMC8517976; doi:10.1021/acs.nanolett.1c02881)
Supplement: Supplementary file 1 — nl1c02881_si_001.pdf [file nl1c02881_si_001.pdf]

Supporting information

Doublet-singlet-doublet transition in a single organic molecule magnet on-surface constructed with up to 3 Aluminum atoms

We-Hyo Soe,[*†‡] Roberto Robles,[*§] Paula de Mendoza,[#] Antonio M. Echavarren,[#] Nicolas Lorente[§Π] and Christian Joachim[†‡]

[†] Centre d'Elaboration de Matériaux et d'Études Structurales (CEMES), Centre National de la Recherche Scientifique (CNRS), Université de Toulouse, 29 Rue J. Marvig, BP 94347, 31055 Toulouse Cedex, France

[‡] International Center for Materials Nanoarchitectonics (WPI-MANA), National Institute for Material Sciences (NIMS), 1-1 Namiki, Tsukuba, Ibaraki 305-0044, Japan

[§] Centro de Física de Materiales CFM/MPC (CSIC-UPV/EHU), Paseo Manuel de Lardizabal 5, 20018 Donostia-San Sebastián, Spain

[#] Institute of Chemical Research of Catalonia (ICIQ), Barcelona Institute of Science and Technology (BIST), 43007 Tarragona, Spain; Department de Química Analítica i Química Orgànica, Universitat Rovira i Virgili, 43007 Tarragona, Spain

[Π] Donostia International Physics Center (DIPC), 20018 Donostia-San Sebastian, Spain

Contents

Suppl. Info. S1: Molecular electronic states; Experiments vs DFT

Suppl. Info. S2: Vibration modes of the Al_3-HATA molecule on surface

Suppl. Info. S1: Molecular electronic states: Experiments vs DFT

As presented in Fig. S1, the changes of the experimental STS electronic resonances energy values and the calculated DFT HATA electronic states show the same trend when the electronic structure of the HATA molecule is modified by coordinating Al ad-atoms one after the other.

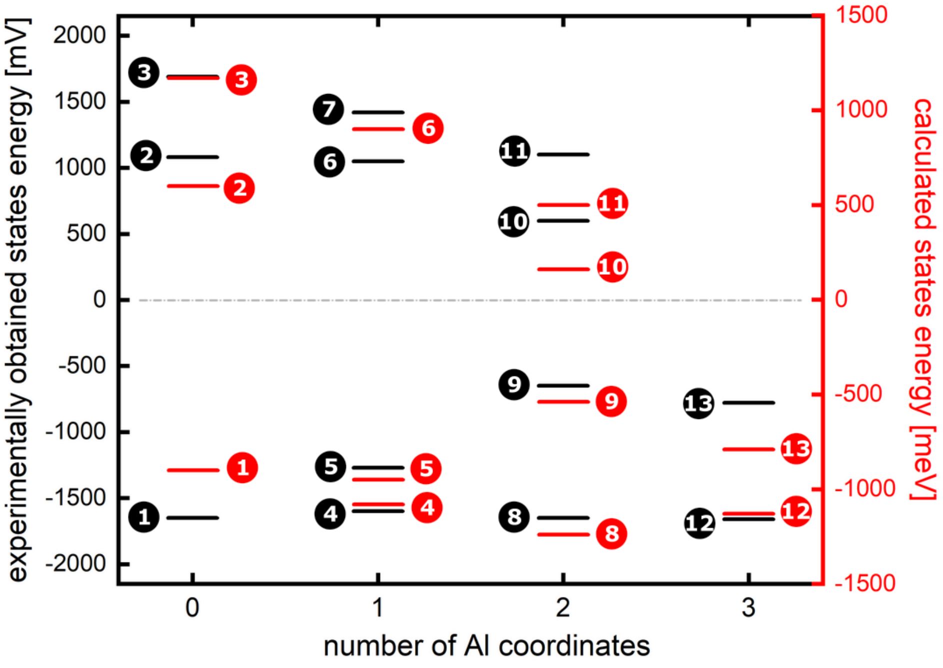

Figure S1. Variations of the experimental STS electronic tunneling resonances as a function of the number of coordinated Al atoms (black) and of the energy position of the DFT calculated HATA electronic state (red). The inserted numbers are related to the Fig. S2 dI/dV mapping below.

The experimental map of each resonance is presented in Fig. S2 with calculated maps. Most of the experimental dI/dV maps are in good agreement with the calculated maps except in the case of the Al_1-complex case. Experimental resonances 4 and 5 appear in a reverse order according to the corresponding calculated maps. Furthermore, calculated electronic state 6 (red) is split in 2 resonances 6 and 7 (black) in the STS experiments. We attribute those differences to the fact that calculating the dI/dV tunneling resonance position in energy using DFT calculation is not reproducing all the details of the many-body character of a given tunneling resonance, for example in the case of an open shell molecular system like the Al_1-complex. As such, this is impacting the calculated maps which are basically the plot of the square of the corresponding calculated DFT molecular orbital (up to a certain density). Experimentally, such a dI/dV mapping can be interpreted as superposing the contribution of many mono-electronic molecular orbitals. This is the case if the many-body molecular electronic state corresponding to a given tunneling resonance

can be effectively decomposed in its molecular orbital mono-electronic components via the corresponding Slater determinants. Via STM tip scanning for performing a dI/dV mapping, such decomposition can lead to a reversal of the experimental maps as compared to the calculated mono-electronic ones calculated with DFT. [1]

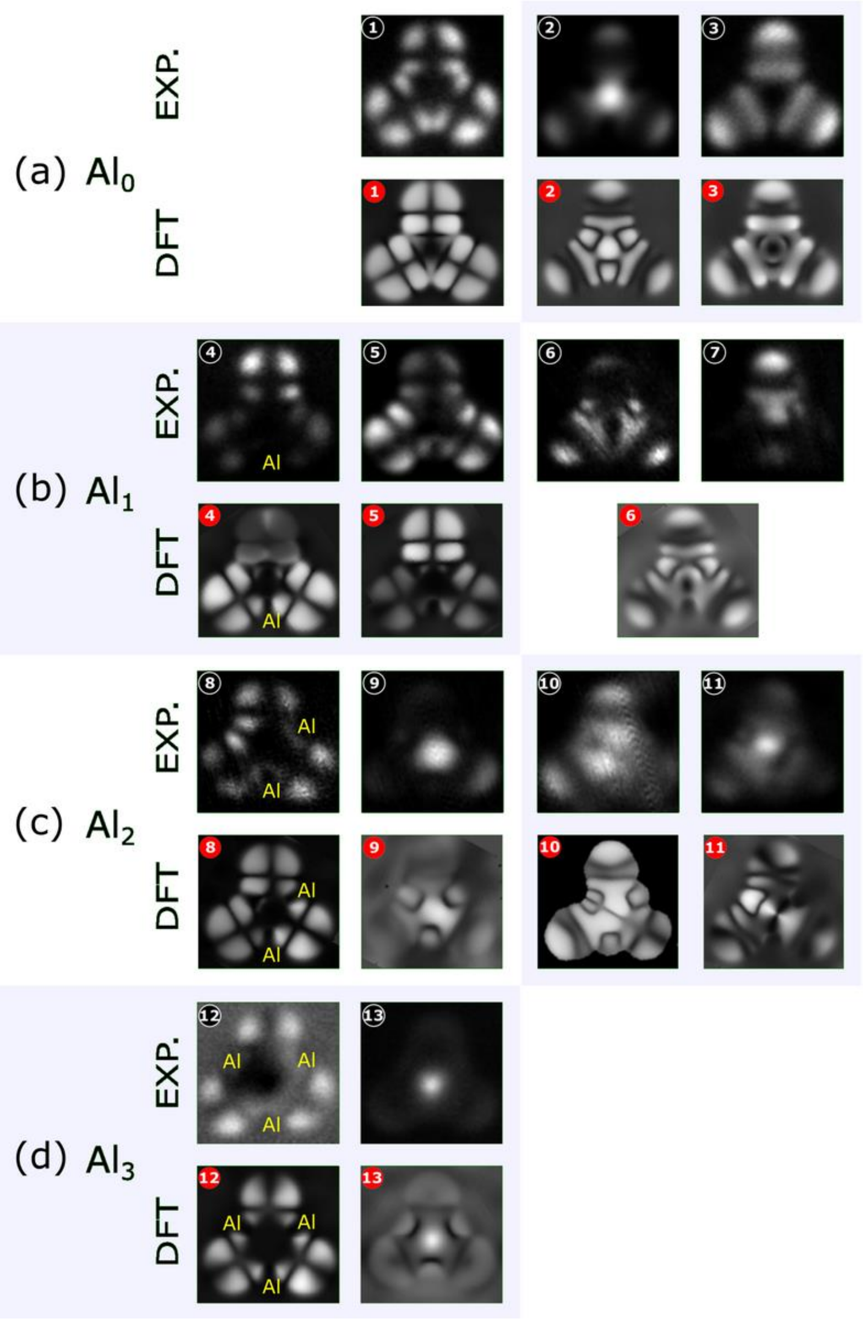

Figure S2. dI/dV maps and calculated STM images correspond to the electronic state positioned on the energy scale in Fig. S1. (a) to (d) are for Al_0- to Al_3-complexes, respectively.

The spin density distributions of Al_1- and Al_3-HATA complexes calculated by DFT in the gas phase are also visualized in Fig. S3. To take electron leakage to the metal substrate into account, neutral and divalent cation molecular states were chosen to produce doublets for Al_1- and Al_3-complexes, respectively, and the Al Cartesian coordinates were extracted from the results of the optimized structure on the slab surface (i.e., Fig. 1(d)) and kept unchanged during calculation.

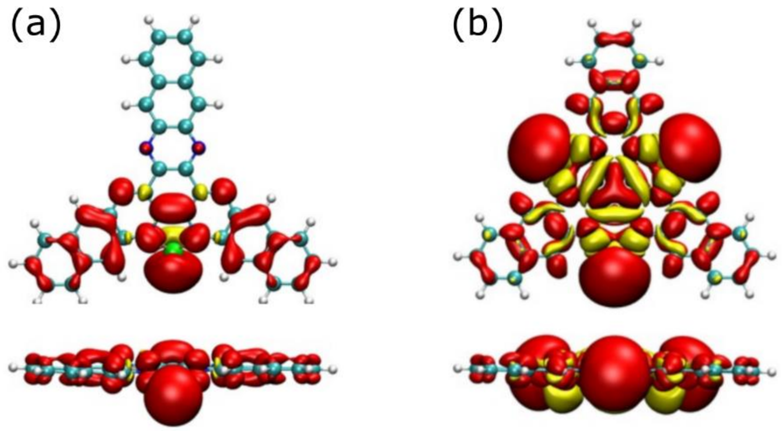

Figure S3. Computed spin density distributions of Al_1- and Al_3-HATA complexes in gas phase using DFT.

Suppl. Info. S2: vibrational modes of the Al_3-HATA molecule on surface

Below about 20 meV, molecular vibrational modes of a 2D planar molecule in gas phase (also in solution) appear as skeleton deformation modes of the entire molecule. Then, the vibrational modes of the local chemical bonds are reaching a maximum energy of about 370 meV for example for the stretching of C-H bond in an organic molecule. In the case of the HATA molecule after the modes corresponding to the soft mode skeleton deformation, the next modes appear to be a combination of a partial skeleton deformation coming from each anthracene chemical group.

When the molecule is adsorbed on a flat surface, vibrational modes perpendicular to the surface are almost suppressed because of repulsion force from the surface. Since the HATA molecule is locally anchored on the surface with a local chemical bond, some of the vibrational modes are shifted in energy.

Fig. S4 is presenting the displacement of the center of mass of an HATA molecule bound to the surface with its three Al ad-atoms in cylindrical coordinates, calculated using DFT. The in-plane

displacement direction is random as shown in Fig. S4(b). The largest center of mass motion appears in-plane as presented in Fig. S4(a). The modes below 10 meV include the largest displacement (very soft mechanical modes) and are in-plane complete HATA molecular skeleton deformations. Perpendicular to the surface complete HATA molecule skeleton deformation modes are arising between 10 meV and 20 meV (Fig. S4(c)). The vibrational modes from 20 meV to 50 meV are combinations of first and second-harmonics due to the partial skeleton deformation of anthracene chemical groups. The modes above 50 meV are by extension combinations of third- and higher-harmonics of the three anthracene branches, finally resulting in an almost aggregation of each mode. In fact, these high-energy modes have less displacement of the center of mass during oscillation, except for two modes indicated by green arrows as shown in Fig. S4(c) and discussed below.

Inelastic electron tunneling spectroscopy (IETS) technique enables molecular vibrations to be detected. In particular, because the STM allows very localized measurements, di-atomic vibrational modes can be clearly detected in well-defined system such as acetylene on Cu(100) [2] and CO on Cu(111).[3] Skeleton deformation vibrations are also observed when the target molecule is confined in its own cluster or in a monolayer. In this case, the molecular motion is restricted (or excited collectively with surrounding molecules).[4,5] Considering these previous studies, it is difficult to capture the molecular skeleton deformation modes on our HATA isolated single molecule. Furthermore, the outer edge of each anthracene branch is a free end for the vibration and the mirror symmetry along the direction of three anthracene branches disappears due to the presence of the surface (see Fig. 1(d)). As a consequence, there is a split in energy into many similar but different vibrational modes. Therefore, the associated high energy vibrational modes are also difficult to be observed. The only vibrational mode of the Al_3-HATA molecule leading to a very distinct vibrational mode is coming from the central core of this molecular complex.

Skeleton deformation modes for the HATA aromatic part (including ν(C=C) modes) are known to be in the range of 160-210 meV.[6,7] In our system, there are many aromatic deformation modes near this energy range due to the presence of various aromatic ring modes in the anthracene branches, and it is difficult to identify which aromatic deformation mode is captured. However, and in Fig. 4(a), we conclude that the higher in energy satellite comes from one of these modes.

The vibrational modes discussed above originate from the HATA molecular core itself. In-plane displacement of center of mass during a vibration is almost the same for molecule alone and with its Al coordinated atoms as confirmed in Fig. S4(a). This means that the Al atoms are not displaced in the horizontal direction because a given Al atom sits on a hollow-site of the fcc (111) surface and in this case a high energy is required to move it horizontally from such potential energy valley. However, this is not the case for the vertical motion of an Al atom. In fact, and as indicated by the green arrows in Fig. S4(c), at around 25 meV only the Al atoms are contributing (light green) while at 51 meV the Al atoms and HATA molecule stretch reciprocally (dark green). For the mode just below 25 meV, there are two modes in very close proximity that have opposite phases to each other. This is related to the vertical vibration of the Al. It probably interferes with the generation of a vibron due to Al vibrations in this energy range. The 51 meV mode is a well-defined symmetric vibrational mode as seen in movie S1, and corresponds to the lower energy satellite peak in Fig. 4(a).

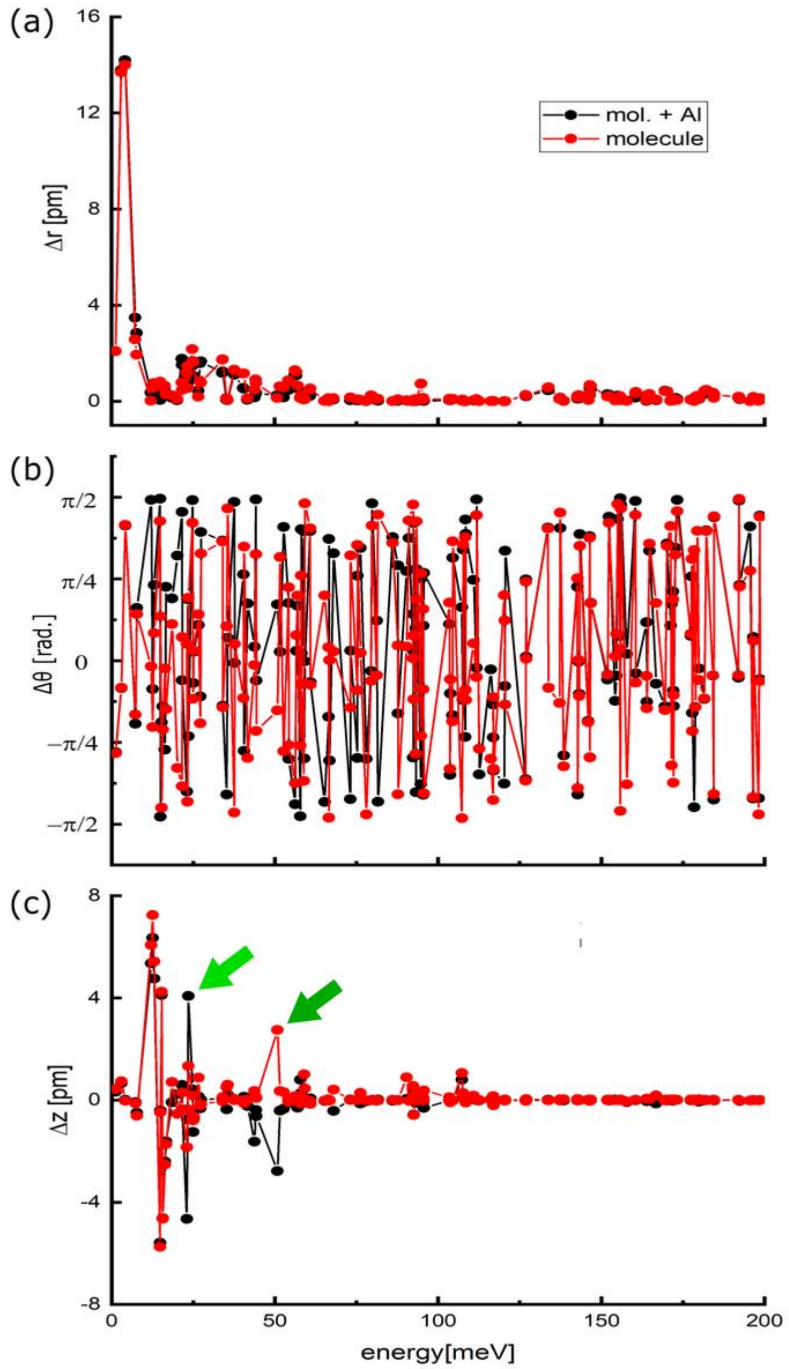

Figure S4. Displacements of the center of mass of the HATA molecule alone (red) and with three Al atoms (black) during its vibrations on the Au(111) surface as calculated by DFT. (a) displacement distance in-plane, (b) displacement angle in-plane, and (c) displacement distance in the direction perpendicular to the surface. Green arrows indicate the modes related to the Al vibrations.

References

(1) Soe, W. -H.; Manzano, C.; De Sarkar, A.; Chandrasekhar, N.; Joachim, C. Direct Observation of Molecular Orbitals of Pentacene Physisorbed on Au(111) by Scanning Tunneling Microscope, *Phys. Rev. Lett.* **2009**, 102, 176102.

(2) Stipe, B. C.; Rezaei, M. A.; Ho, W. Single-Molecule Vibrational Spectroscopy and Microscopy, *Science* **1998**, 280 1732-1735.

(3) Vitali, L.; Ohmann, R.; Kern. K. Surveying Molecular Vibrations during the Formation of Metal-Molecule Nanocontacts, *Nano Lett.* **2010**, 10 657-600.

(4) Eickhoff, F.; Kolodzeiski, E.; Esta, T.; Fournier, N.; Wagner, C.; Deilmann, T.; Temirov, R.; Rohlfing, M.; Tautz, F. S.; Anders, F. B. Inelastic Electron Tunneling Spectroscopy for Probing Strongly Correlated Many-Body Systems by Scanning Tunneling Microscopy, *Phys. Rev.* ***B*** **2020**, 101 125405.

(5) Wegner, D.; Yamachika, R.; Zhang, X.; Wang, Y.; Crommie, M. F.; Lorente, N. Adsorption Site Determination of a Molecular Monolayer via Inelastic Tunneling, *Nano Lett.* **2013**, 13, 2346-2350.

(6) Salhani, C.; Della Rocca, M. L.; Bessis, C.; Bonnet, R.; Barraud, C. Lafarge, Pl; Chevillot, A.; Martin, P.; Lacroix, J.-C. Inelastic Electron Tunneling Spectroscopy in Molecular Junctions Showing Quantum Interference, *Phys. Rev.* ***B*** **2017**, 95, 165431.

(7) Wang, S. Intrinsic Molecular Vibration and Rigorous Vibrational Assignment of Benzene by First-Principle Molecular Dynamics, *Sci. Rep.* **2020**, 10, 17875.
